# MRI markers of brain network integrity relate to neurological outcome in postanoxic coma

**DOI:** 10.1016/j.nicl.2022.103171

**Published:** 2022-08-26

**Authors:** Hanneke M. Keijzer, Puck A.M. Lange, Frederick J.A. Meijer, Bart A.R. Tonino, Michiel J. Blans, Catharina J.M. Klijn, Cornelia W.E. Hoedemaekers, Jeannette Hofmeijer, Rick C. Helmich

**Affiliations:** a Department of Neurology, Rijnstate Hospital, 6800 TA Arnhem, the Netherlands; b Department of Neurology, Donders Institute for Brain, Cognition, and Behaviour, Radboud University Medical Centre, 6500 HC Nijmegen, the Netherlands; c Department of Medical Imaging, Radboud University Medical Centre, 6500 HC Nijmegen, the Netherlands; d Department of Radiology, Rijnstate Hospital, 6800 TA Arnhem, the Netherlands; e Department of Intensive Care Medicine, Rijnstate Hospital, Arnhem, the Netherlands; f Department of Intensive Care Medicine, Radboud University Medical Centre, 6500 HC Nijmegen, the Netherlands; g Department of Clinical Neurophysiology, University of Twente, Faculty of Science and Technology, 7522 NB Enschede, the Netherlands

**Keywords:** Postanoxic coma, Functional MRI, Diffusion Weighted Imaging, Cardiac arrest, CPC, Cerebral Performance Category, ICU, Intensive Care Unit, DMN, Default Mode Network, SN, Salience Network, ECN, Executive Control Network, FPN, Frontoparietal Network, GCS, Glasgow Coma Scale, GPD, Generalized Periodic Discharges, WLST, Withdrawal of Life Sustaining Therapy, MD, Mean Diffusivity

## Abstract

**Aim:**

Current multimodal approaches leave approximately half of the comatose patients after cardiac arrest with an indeterminate prognosis. Here we investigated whether early MRI markers of brain network integrity can distinguish between comatose patients with a good versus poor neurological outcome six months later.

**Methods:**

We performed a prospective cohort study in 48 patients after cardiac arrest submitted in a comatose state to the Intensive Care Unit of two Dutch hospitals. MRI was performed at three days after cardiac arrest, including resting state functional MRI and diffusion-tensor imaging (DTI). Resting state fMRI was used to quantify functional connectivity within ten resting-state networks, and DTI to assess mean diffusivity (MD) in these same networks. We contrasted two groups of patients, those with good (n = 29, cerebral performance category 1–2) versus poor (n = 19, cerebral performance category 3–5) outcome at six months. Mutual associations between functional connectivity, MD, and clinical outcome were studied.

**Results:**

Patients with good outcome show higher within-network functional connectivity (fMRI) and higher MD (DTI) than patients with poor outcome across 8/10 networks, most prominent in the default mode network, salience network, and visual network. While the anatomical distribution of outcome-related changes was similar for functional connectivity and MD, the pattern of inter-individual differences was very different: functional connectivity showed larger inter-individual variability in good versus poor outcome, while the opposite was observed for MD. Exploratory analyses suggested that it is possible to define network-specific cut-off values that could help in outcome prediction: (1) high functional connectivity and high MD, associated with good outcome; (2) low functional connectivity and low MD, associated with poor outcome; (3) low functional connectivity and high MD, associated with uncertain outcome.

**Discussion:**

Resting-state functional connectivity and mean diffusivity-three days after cardiac arrest are strongly associated with neurological recovery-six months later in a complementary fashion. The combination of fMRI and MD holds potential to improve prediction of outcome.

## Highlights

• Functional connectivity and MD are significantly higher in patients with good outcome post cardiac arrest.
• Posterior brain areas appear to be particularly vulnerable to hypoxia.
• fMRI and DTI appear to be complimentary for outcome prediction after cardiac arrest.

## 1 Introduction

Cardiac arrest is a major cause of death and disability, affecting 27–91 per 100,000 of the European population yearly (Grasner et al., 2020). Of all patients, 7–43 % arrive at the hospital alive after successful cardiopulmonary resuscitation (Grasner et al., 2020). Most of these patients are admitted to an intensive care unit (ICU) in a comatose state. Approximately 50 % of patients never regain consciousness or remain severely disabled as a result of hypoxic-ischemic brain injury (Bongiovanni et al., 2020, Ruijter et al., 2019). Reliable early prediction of neurological outcome is important to optimize care for patients and counselling of their relatives. The current European post-resuscitation guideline recommends a multimodal approach for prediction of a poor outcome, including clinical examination, electrophysiology, serum biomarkers, and neuroimaging (Cronberg et al., 2020, Nolan et al., 2021). Together, these measures can reliably predict outcome in 30–50 % of the patients (Bongiovanni et al., 2020, Moseby-Knappe et al., 2020, Ruijter et al., 2019). Therefore, new additional biomarkers for early prognosis are highly needed.

Here, we focus on functional magnetic resonance imaging (fMRI), which indirectly measures neuronal activity using the blood oxygenation level-dependent (BOLD) signal. It is well-established that the brain is organized into spatially distinct cerebral networks, by which various brain regions are functionally and anatomically connected (Damoiseaux et al., 2006). These various resting state networks can be identified by assessing inter-regional coupling during a brief fMRI scan in the absence of a task (typically 5–10 min). In various neurological and psychiatric disorders, the magnitude of resting-state functional connectivity has been taken as a measure of network integrity, raising the possibility to use fMRI as a biomarker of brain (dys)function (Rosazza and Minati, 2011).

Analysis of fMRI derived resting state networks holds potential to add to outcome prediction of comatose cardiac arrest survivors. Specifically, EEG analyses have shown that measures of brain activity are highly sensitive to hypoxic-ischemic brain damage (Ruijter et al., 2019, Spalletti et al., 2016, Westhall et al., 2016). fMRI may add to EEG, because it is sensitive not only to cortical but also to subcortical brain activity and has a higher spatial resolution. Furthermore, various resting state networks have been shown to play a crucial role in disorders of consciousness. Especially the default mode network (DMN), executive control network (ECN), frontoparietal network (FPN), and salience network (SN) have been identified as relevant brain networks for maintaining consciousness (Demertzi et al., 2014, Di Perri et al., 2014, Hannawi et al., 2015, Heine et al., 2012, Qin et al., 2015).

Previous studies on fMRI for prediction of outcome after cardiac arrest showed decreased connectivity in patients with poor outcome, but most studies were small (n = 13–90), performed MRI at various times after cardiac arrest (3–12 days) and none of the studies reported predictive values in addition to current clinical care (Koenig et al., 2014, Norton et al., 2012, Sair et al., 2017, Wagner et al., 2020).

Previously, also diffusion weighted imaging (DWI) MRI has been proposed as a predictor of outcome (Hirsch et al., 2020, Nolan et al., 2021, Vanden Berghe et al., 2020) and was complementary to EEG measures of brain activity in previous studies (Beuchat et al., 2020, Bevers et al., 2018). This imaging technique relies on random motion of water molecules, which can be restricted as a result from e.g. cytotoxic oedema. Extensive DWI abnormalities were associated with poor outcome, but not all patients with severe global ischemic brain injury and functional deficits presented with DWI abnormalities (Beuchat et al., 2020). Combining DWI for detection of oedema with EEG and fMRI measuring neuronal activity could potentially improve outcome prediction in postanoxic coma.

Here we performed a prospective cohort study in patients who remained comatose at hospital admission after cardiac arrest, where resting-state fMRI and Diffusion tensor imaging (DTI) was performed within 2–4 days after cardiac arrest. We adopted a multi-modal approach, testing the hypothesis that resting state functional connectivity and mean diffusivity are reduced in patients with poor outcome. In addition, we assessed mutual associations between network strengths, mean diffusivity, and outcome at six months, to establish relations between functional connectivity within resting state networks and diffusion restriction in brain areas belonging to these networks.

## 2 Materials and methods

### 2.1 Study design

We performed a multicentre cohort study in two Dutch hospitals, Rijnstate hospital (Arnhem) and Radboud university medical centre (Radboudumc, Nijmegen). We included patients between June 2018 and October 2020. The work described has been carried out in accordance with The Code of Ethics of the World Medical Association (Declaration of Helsinki) for experiments involving humans, and the study protocol was approved by the Committee on Research Involving Human Subjects region Arnhem-Nijmegen and registered on clinicaltrials.gov (identifier: NCT03308305).

### 2.2 Study population

Patients were included within 72 h after cardiac arrest after legal representative’s consent. Inclusion criteria were: minimum age of 18, Glasgow Coma Scale (GCS) ≤ 8 at admission, cardiac arrest based on cardiac cause or pulmonary embolism and admission to the Intensive Care Unit (ICU). Exclusion criteria included: pregnancy, life expectancy < 24 h after admission, known progressive neurological disease (e.g. brain tumour or neurodegenerative disease), contraindication for MRI scanning (e.g. presence of pacemaker, neurostimulator, etc.) and pre-existent dependency in daily living.

Patients were treated according to international guidelines and local protocols. This included targeted temperature management, induced as soon as possible after arrival at the ICU and maintained for 24 h (36 °C at Rijnstate hospital, 32–34 °C at Radboudumc). Thereafter, controlled passive rewarming occurred at a speed of 0.25–0.5 °C per hour, followed by active normothermia maintenance. Sedation and analgesia mainly consisted of propofol, midazolam, morphine and/or sufentanil.

Withdrawal of life sustaining therapy (WLST) was considered ≥ 72 h after cardiac arrest, when patients were off sedation and had reached normothermia. Decisions on WLST were based on European guidelines, including bilateral absence of somatosensory evoked potentials (SSEPs), treatment-resistant myoclonus, and incomplete return of brainstem reflexes (Nolan et al., 2021). The Dutch guideline “prognosis after postanoxic coma” includes EEG as a prognostic tool since April 2019. This guideline states that a poor outcome is likely in case of an isoelectric EEG > 12 hr, or a low voltage or suppression EEG with identical bursts or generalized periodic discharges (GPD) > 24 hr. Decision-making regarding WLST always considered the specific background of the patient and involved consultation of a multidisciplinary team and the patient’s family. In case of doubt, the decision was postponed and the patient was re-evaluated at a later time. The treatment team was blinded to the MRI measures published here.

### 2.3 Outcome

The primary outcome measure was neurological outcome, operationalized as the Cerebral Performance Category (CPC-score) at six months after cardiac arrest, dichotomized as good (CPC 1–2, no/mild neurological impairment) and poor (CPC 3–5, severe neurological damage, vegetative state or death). A standardized telephone interview based on the EuroQol-6D questionnaire was used to assess CPC scores.

### 2.4 Data acquisition

All patients were scanned using a 3 Tesla MRI scanner on either Philips Ingenia (Rijnstate) or Siemens Skyra (Radboudumc) at 2–4 days after cardiac arrest. Resting-state fMRI was acquired using a gradient-echo echo planar imaging sequence (Philips: TE/TR 27/2220 ms, voxel size 3.0 * 3.0 * 3.0 mm, 220 volumes; Siemens: TE/TR 27/2280 ms, voxel size 3.2 * 3.2 * 3.0 mm, 220 volumes). Anatomical data were acquired using 3D-T1 (Philips: 3D TFE, TE/TR 3.8/8300 ms, voxel size 1.0 * 1.0 * 1.0 mm; Siemens: MPRAGE, TR/TE 3.41/2400 ms, voxel size 0.9 * 0.9 * 1.0 mm). Diffusion imaging was acquired by a DTI sequence, using a 2D echo-planar imaging sequence (Philips: TR/TE 9000/95 ms voxel size 2.0 * 2.0 * 2.0 mm, 1 unweighted image, 32 images with a b-value of 1000 s/mm^2^; Siemens: TR/TE 9700/95 ms, voxel size 2.0*2.0*2.0 mm, 1 unweighted image, 30 images with a b-value of 1000 s/mm^2^).

### 2.5 Data analyses

#### 2.5.1 fMRI

Individual reports of MRI Quality Control MRIqc (Esteban et al., 2017) were visually assessed before entering the preprocessing pipeline. Preprocessing was performed using FMRIPREP version 20.0.6 (Esteban et al., 2019), which included motion correction, resampling to the MNI152NLin6cAsym standard space, removal of non-steady state volumes, spatial smoothing with an isotropic, Gaussian kernel of 6 mm full-width half-maximum (FWHM) and non-aggressive denoising based on independent component analysis (ICA-AROMA) (Pruim et al., 2015), see Supplementary section 1 for a detailed overview. Data quality of FMRIPREP’s output was visually checked. Next, additional spatial smoothing was applied to achieve net smoothing of 8 mm FWHM. The identified CSF and WM nuisance regressors were regressed out of the non-aggressive AROMA denoised files to exclude physiological noise. We removed the first five volumes to ensure a steady state, and applied a high-pass filter of 0.007 Hz.

Preprocessed data were submitted to probabilistic independent component analysis (ICA) on a group level, using FSL’s MELODIC (Beckmann and Smith, 2004). This decomposed the BOLD data into 20 group-average spatially independent components. We were primarily interested in resting state networks previously associated with consciousness, i.e. the DMN, ECN, FPN, and SN. For explorative reasons, we extended our analyses to all other networks identified by the ICA.

For each subject, we regressed the group level spatial map to individual spatial maps of the identified resting state networks, using FSL’s Dual Regression (Beckmann et al., 2009). Study site and framewise displacement were included as covariates. The resulting maps represent voxel-wise connectivity strength for each patient. We calculated mean normalized connectivity strength of the identified networks for each patient, based on the z-stat output of dual regression.

#### 2.5.2 Mean diffusivity

Preprocessing of the DTI data consisted of denoising, removal of Gibbs artefacts (MRtrix version 3.0, https://www.mrtrix.org) and correction for Eddy current distortions and motion (Andersson and Sotiropoulos, 2016). Free water elimination was applied using the free water imaging toolbox to calculate free-water corrected mean diffusivity (MD) (Pasternak et al., 2009). Because of free water elimination, no further thresholding was necessary to correct for CSF in cortical and *peri*-ventricular areas. We nonlinearly transformed the MD images to MNI152 standard space to be analogous to the functional images. We created spatial masks for each of the identified functional networks based on the components identified by FSL melodic (threshold F ≥ 3.2) and calculated the average MD within each network per subject.

#### 2.5.3 Relation with the EEG

We related the MRI results to the EEG at 12 and 24 hr after cardiac arrest, to assess potential complementarity of MRI and EEG in outcome prediction. We scored 5-minute EEG epochs as previously described (Ruijter et al., 2019), blinded for patient outcome. Epochs were classified as suppressed, synchronous bursts or identical bursts with suppressed background activity, GPDs on suppressed background activity, low voltage patterns, epileptiform activity, heterogeneous burst suppression (50–90 % suppression), discontinuous patterns (10–49 % suppression), or continuous activity (<10 % suppression).

### 2.6 Statistical analyses

Continuous data are presented as mean and standard deviation (SD) in case of normal distribution, or median [interquartile range (IQR)] otherwise. We used chi-squared tests for ordinal and student t-tests of Mann Whitney-U tests for continuous variables to compare groups with good and poor outcome. Effect sizes were calculated using Cohen’s d.

To study between-group differences in functional connectivity, we compared mean normalized functional connectivity for the identified resting state networks, corrected for study site and framewise displacement using ANCOVA analysis. Between group differences in MD were corrected for study site using ANCOVA. We corrected for multiple comparisons (i.e. multiple brain networks) using false discovery rate (FDR) (Benjamini and Yekutieli, 2001). Corrected p-values ≤ 0.05 were assumed statistically significant. Statistical analyses were performed using R version 3.5.3 or MATLAB R2021a.

Of the networks with the highest effect sizes of fMRI and MD (Cohen’s d > 1), we explored the relation between connectivity strength and MD using scatterplots of individual values for connectivity and strength, grouped by outcome. To indicate a potential predictive value, we established preliminary cut off values by eyeballing of the results. These cut off values were aimed to predict poor outcome at 100 % specificity. ROC analyses were not performed because of the relatively small cohort size and the lack of an independent validation cohort. We compared the cut off values found here to previous established cut off values indicated by studies on ADC imaging (Hirsch et al., 2020, Keijzer et al., 2022, Wouters et al., 2021).

### 2.7 Post hoc analyses

We performed three post hoc analyses to gain further insight in the possible predictive value of functional MRI. To prevent an expansion of the number of analyses, we restricted these post-hoc analyses to the three brain networks with the largest group difference between good and poor outcome (Cohens’ d > 1: DMN, SN, and visual network).

At the time of MRI scanning at day two to four, a subset of the patients regained consciousness and were awake during MRI. In the first post-hoc analysis, we compared within network connectivity, corrected for MD and study site for patients who were comatose at the time of MRI. For these patients, prognosis was uncertain at time of MRI and the level of consciousness was comparable between subjects.

For the second post hoc analysis, we compared within-network functional connectivity between patients with a good versus poor outcome, while controlling for all predictors of a poor outcome that are currently included in the European guideline: bilaterally absent N20 on SSEP, absent pupillary or corneal reflexes ≥ 72 hr, and a highly malignant EEG pattern (Nolan et al., 2021) within the DMN, SN, and visual network. With these analyses, we therefore estimated the potential of fMRI to add to prediction based on ‘classical’ predictors.

The third post-hoc analysis aims to indicate the potential additional predictive value of fMRI to MD imaging. To this end, we repeated our analyses in a subgroup of patients with an MD > the threshold predicting poor outcome (i.e. patients without abnormal MD findings). Within this subgroup, we performed group comparisons using Mann Whitney-U tests (good versus poor outcome) on functional connectivity within each network.

## 3 Results

### 3.1 Subjects

Baseline characteristics of the 48 included patients are listed in Table 1. We screened 261 patients for eligibility and included 64 patients. Main reasons why patients were not included were no permission from the legal representative (n = 48) and a non-cardiac cause of the arrest (n = 44, see Supplementary Fig. 1). Subsequently, 14 patients were excluded because MRI could not be obtained at three days (±one) after cardiac arrest: four were haemodynamically unstable, four died or were transferred to another hospital before MRI, one was tested positive for COVID-19, hampering transport to the radiology department, and five had other organizational reasons (Supplementary Fig. 1). In addition, one patient was excluded because of inadequate fMRI image quality and another because of a recent cerebral infarction.

**Table 1:** Baseline characteristics of the included patients with good and poor outcome six months after cardiac arrest.

Characteristic	Good outcome (n = 29)	Poor outcome (n = 19)	P-value
Age (year)	56 (11)	66 (11)	<0.01*
Male	24 (83)	13 (68)	0.24
OHCA	29 (1 0 0)	19 (100 %)	NA
Shockable first rhythm	29 (1 0 0)	14 (74)	<0.01*
Duration of resuscitation (min)	12 [10–15]	23 [20–33]	<0.01*
Absent pupillary light reflex ≥ 72 hr	0 (0)	4 (21)	<0.01*
Motor score ≤ 3 at 72 hr	1 (3)	14 (74)	<0.01*
Bilaterally absent SSEP response ≥ 72 hr	0 (0)	4 (21)	<0.01*
Time ROSC-MRI (hour)	78 (24)	77 (27)	0.97
Comatose during MRI	10 (36 %)	18 (95 %)	<0.01*
Treatment with sedatives during MRI (*propofol, midazolam, dexmedetomidine*)	10 (34 %)	17 (89 %)	<0.01*
Treatment with opiates during MRI (*morphine, sufentanil, remifentanil*)	4 (14 %)	5 (26 %)	0.28
Survival till discharge from primary hospital	29 (1 0 0)	4 (21)	<0.01*
CPC score at 6 months			
- 1	13 (45)		
- 2	16 (55)		
- 3		3 (16)	
- 4		0 (0)	
- 5		16 (84)	

Dichotomous variables are listed as n (%). Continuous variables are listed as mean (standard deviation) in case of normal distribution or median [IQR] otherwise. Group differences are calculated using t-tests, Man-Whitney U or chi-square tests. Significant differences are indicated by *. CPC: cerebral performance category; OHCA: out of hospital cardiac arrest; ROSC: return of spontaneous circulation.

**Fig. 1:**
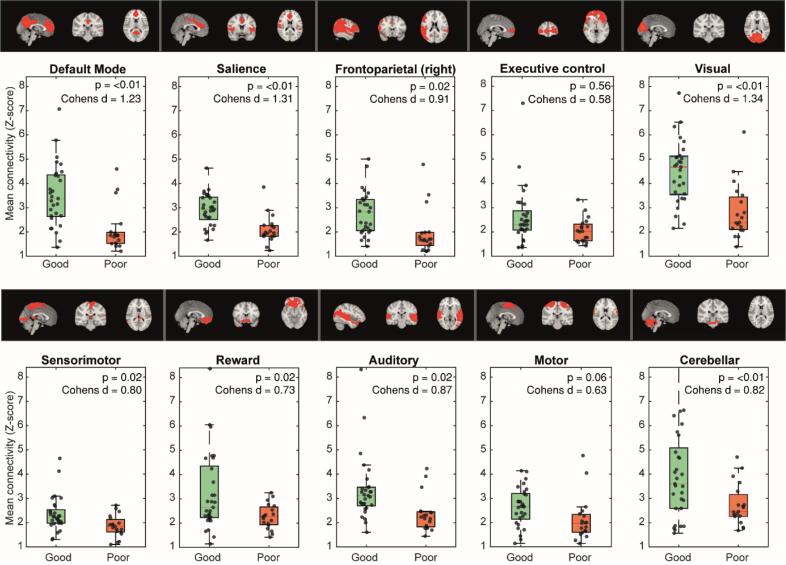
Spatial maps and mean functional connectivity within the ten identified resting state networks per subject, grouped by outcome. P-values are corrected for study site and framewise displacement. Correction for multiple comparisons was applied using the false discovery rate (Benjamini and Yekutieli, 2001).

### 3.2 MRI analyses

We identified ten spatially independent components as resting-state networks, while ten other networks were judged artefactual. The topographies of the ten identified resting state networks were consistent with the Default Mode (DMN), Salience (SN), right Frontoparietal (RFPN), Executive Control (ECN), Visual (VN), Sensorimotor, Reward, Auditory, Motor, and Cerebellar network (Damoiseaux et al., 2006) (Fig. 1).

**Fig. 2:**
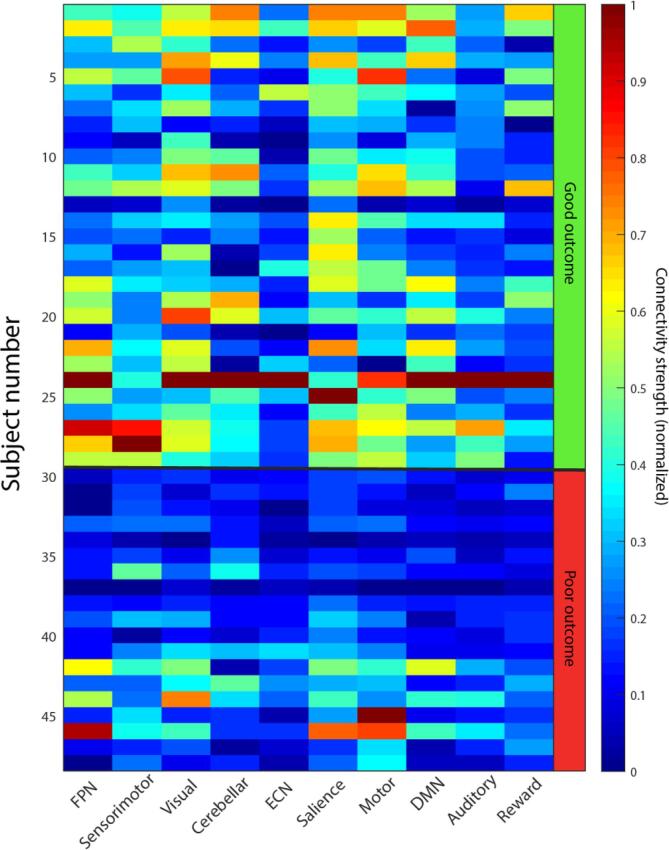
Normalized connectivity strength for individual patients across networks, grouped by outcome. High or low connectivity appears to be consistent across the different resting state networks. DMN: default monde network; ECN: Executive control network; FPN: frontoparietal network.

Patients with good outcome showed significantly higher (p < 0.05, FDR corrected) functional connectivity in all resting state networks except the ECN and motor network than patients with poor outcome, with a medium to large effect size (Cohen’s d: 0.58–1.34, Fig. 1). The level of connectivity was consequent across networks for the individual patients (Fig. 2). Of the four networks previously associated with consciousness (DMN, SN, ECN and FPN), the SN showed the highest effect size (Cohen’s d = 1.31, Fig. 1). Of all ten identified networks, the visual network showed the largest effect size (Cohen’s d = 1.34). Study site was a significant covariate in all networks except the DMN and ECN. This effect was not explained by a difference in outcome between sites (chi-square test, p = 0.34). Framewise displacement was never a significant covariate (Supplementary Table 1).

In the boxplots of Fig. 1, we considered three subjects in the poor outcome group as outliers, with higher functional connectivity in especially the DMN and FPN, as compared to the other patients with poor outcome. Of these three patients, two survived with a CPC score of 3 (severe disability), but regained some possibilities for communication and mobility*.* The third was an elderly patient who primarily recovered to a GCS of 13, but died after the decision of WLST based on multiple comorbidities. In the other patients with poor outcome, one survived with a CPC of 3, the others died after WLST.

#### 3.2.1 Mean diffusivity

**Fig. 3:**
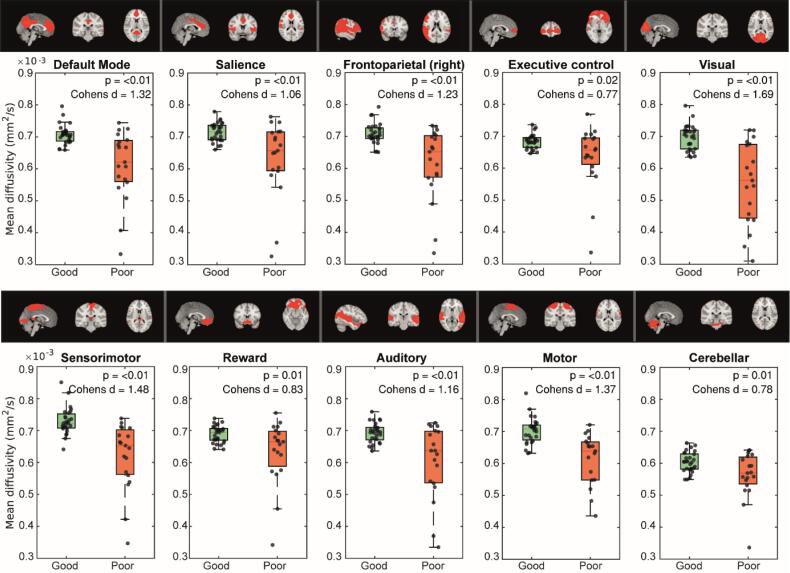
Spatial maps and average mean diffusivity within the location of the identified resting state networks per subject, grouped by neurological outcome. P-values are corrected for study site. Correction for multiple comparisons was applied using the false discovery rate (Benjamini and Yekutieli, 2001).

Mean diffusivity in the various resting state networks was significantly lower in patients with poor versus good neurological outcome (p < 0.05, FDR corrected; Fig. 3) with a medium to large effect size (Cohen’s d: 0.77–1.69). The largest effect size was found in the visual network (Cohen’s d 1.69, Fig. 3). Study site was a significant covariate in all group comparisons, although scan protocols were harmonized between centres (Supplementary Table 2).

#### 3.2.2 Relationship between functional connectivity and MD

**Fig. 4:**
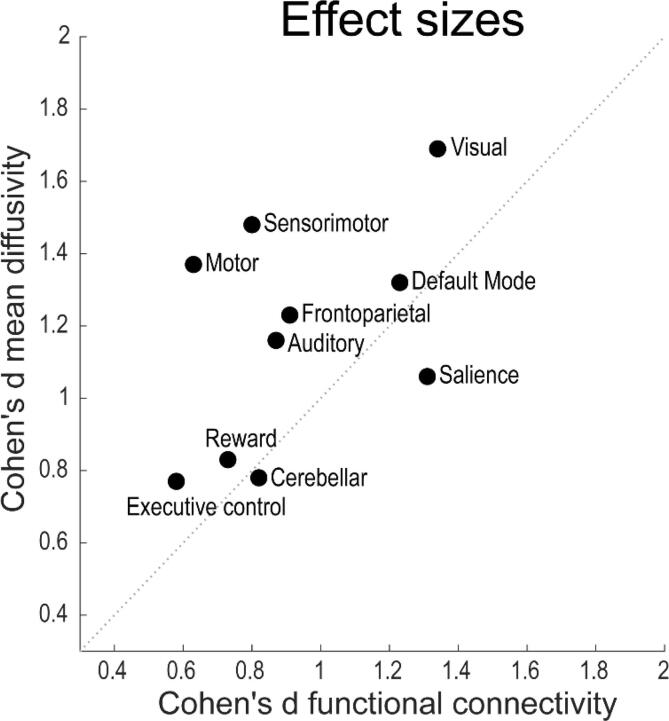
Effect sizes, represented as Cohen’s D, for mean diffusivity against the effect sizes of functional connectivity. Networks with high effect size of functional connectivity typically also show high effect sizes of mean diffusivity.

Networks with high effect sizes in functional connectivity typically also showed high effect sizes in MD (Fig. 4). Among the ten networks, particularly the DMN, SN and visual network showed a large effect size (Cohen’s d > 1) for both modalities.

On the other hand, when comparing the pattern of inter-individual variability for the two MRI modalities, we found clear differences: functional connectivity has larger inter-individual variability (i.e. large IQR) in patients with good versus poor neurological outcome (Fig. 1), whereas MD has larger inter-individual variance in patients with poor outcome vs patients with good outcome (Fig. 3). In other words, the IQR representing functional connectivity of the good outcome group has limited overlap with the IQR representing the poor outcome group, and vice versa for MD. This pattern was consistent across all ten networks. This suggests that fMRI may be better suited to predict a good outcome, whereas MD seems better to predict a poor outcome.

**Fig. 5:**
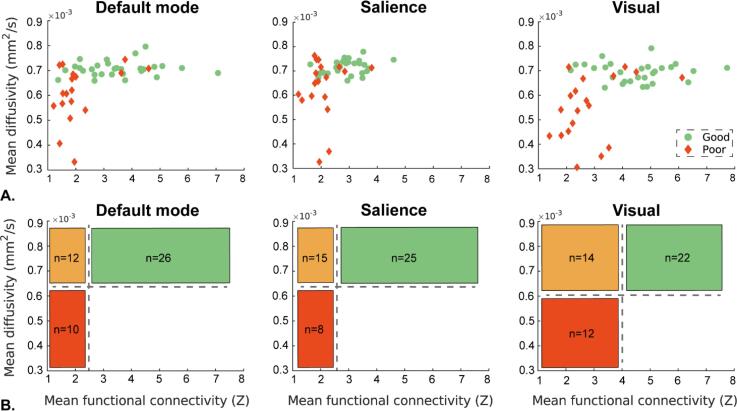
A: mean normalized mean diffusivity (MD) against functional connectivity of the three networks with Cohen’s D > 1 for both fMRI and MD, for patients with good and poor neurological outcome. B: global relation of mean diffusivity and functional connectivity in relation to outcome, and the number of subjects in each category. Green: associated with good outcome, orange: associated with indeterminate outcome, red: associated with poor outcome. Thresholds are based on eyeballing of the scatterplots.

Fig. 5A illustrates inter-individual variability in the relationship between functional connectivity and MD in more detail for the DMN, SN, and visual network. Visual inspection of the scatterplots of Fig. 5A suggests that it may be possible to define cut-off values that could help in outcome prediction. Within our cohort, functional connectivity within the DMN and SN with z > 2,5 and MD > 650*10^−6^ mm^2^/s indicated a good outcome, whereas z < 2,5 and MD < 650*10^−6^ mm^2^/s indicated a poor outcome. For the visual network, the thresholds were z > 4 and MD > 600*10^−6^ mm^2^/s for good outcome, and z < 4,0 and MD < 600*10^−6^ mm^2^/s for poor outcome. MD thresholds predicting a poor outcome correspond with previous studies on ADC imaging of comatose patients after cardiac arrest (Hirsch et al., 2020, Keijzer et al., 2022, Wouters et al., 2021). This leaves an intermediate group with MD > threshold and functional connectivity < threshold. The different prediction-groups based on these thresholds are displayed in Fig. 5B, where red boxes indicate patients with a poor outcome, green boxes patients with a good outcome, and orange boxes patients with an indeterminate outcome.

Of the 29 patients with good outcome, 26 were in the green box with high functional connectivity and high MD of at least one of the three networks depicted in Fig. 5 (DMN, SN and visual network), the other three patients were in the orange box, with low functional connectivity and high MD, of all three networks. Of the 19 patients with poor outcome, 13 were in the red box, indicating low functional connectivity and low MD, of at least one of these three networks. There were no patients with good outcome in the red box. Three patients with poor outcome were in the green box, and three in the orange box of all three networks. In total, 25 subjects, of whom nine with poor outcome, were placed in this indeterminate category of at least one of the three networks. No patients had high MD in combination with low functional connectivity.

#### 3.2.3 Relationship between functional connectivity, MD and EEG

Patients with high functional connectivity and high MD in the DMN, SN and/or visual network (green; n = 26) typically showed continuous (n = 19) or discontinuous and heterogeneous burst suppression (n = 7) EEG at 12 and 24 h, defined as EEG with<50 % suppression. Patients with low functional connectivity and low MD (red; n = 13) had divergent, but often disturbed, EEG patterns: six had synchronous bursts on a suppressed background at 12 hr, two showed epileptiform activity and three had heterogeneous burst suppression at 12 and 24 hr. Only one subject in the red box showed continuous EEG activity at 12 and 24 hr and one had no EEG registration. Out of the patients with low functional connectivity and high MD in all three networks (orange; n = 6), three with good outcome had continuous or discontinuous EEG patterns at 12 and 24 hr. Three patients with poor outcome had severely disturbed EEG: one low voltage pattern, one synchronous burst on a suppressed background, and one GPDs on a suppressed background.

### 3.3 Post hoc analyses

We performed three post-hoc analyses. First, in patients who were comatose at time of MRI (n = 29; 18 with poor outcome), functional connectivity within the DMN and SN remained significantly higher in patients with good vs poor outcome (p = 0.01 and < 0.01 respectively), whereas the visual network was no longer significantly different (p = 0.06). See Supplementary Table 3 for a complete overview. Second, after correction for absent SSEPs, absent pupillary or corneal reflexes, and highly malignant EEG, functional connectivity within the DMN, SN, and visual network remained significantly higher for patients with good than poor outcome (p = 0.03, <0.01, 0.01 respectively). None of the ‘classical’ predictors was marked as a significant covariate by the ANCOVA analyses (Supplementary Table 4).

Third, we performed subgroup analyses in patients with MD > 650*10^−6^ mm^2^/s of the DMN and salience network and MD > 600*10^−6^ mm^2^/s for the visual network. We established these cut-off values by eyeballing the scatterplots of Fig. 5, but they correspond with previous studies using ADC imaging (Hirsch et al., 2020, Keijzer et al., 2022, Wouters et al., 2021). This resulted in a subset of 36–40 patients, of whom 29 with a good outcome. In line with our hypothesis, we found that significant group differences remained between patients with good and poor outcome and MD > threshold for the DMN and salience network, but not for the visual network (Supplementary Table 5). This suggests that fMRI is complimentary to MD imaging in the DMN and salience network.

## 4 Discussion

This study has three main findings. First, functional connectivity and mean diffusivity within large-scale resting state networks are significantly higher in patients with good versus poor neurological outcome at six months. These group differences were present in 8 of 10 resting state networks. Second, effect sizes were larger for resting state networks that are located in posterior brain regions (i.e. DMN, visual network), both for functional connectivity and for MD. Third, we found larger inter-individual differences in patients with good versus poor neurological outcome for fMRI, but for poor vs good outcome for MD. These results support the hypothesis that resting state functional connectivity and mean diffusivity at three days after cardiac arrest are associated with good or poor neurological outcome at six months in a complementary way.

### 4.1 Resting-state networks in coma after cardiac arrest

In the resting state functional connectivity analyses, our main outcome measure was the degree of inter-regional coupling within several intrinsic cerebral networks, based on spontaneous fluctuations in the BOLD response. We have shown that high values of functional connectivity in almost all resting state networks are associated with a good neurological outcome. Decreased functional connectivity was seen in patients with either good or poor outcome, and therefore not invariably associated with a poor outcome.

These results are in line with previous studies, that showed decreased connectivity in patients with poor outcome, especially within the DMN (Koenig et al., 2014, Norton et al., 2012, Sair et al., 2017, Wagner et al., 2020). However, most of these studies applied a large time interval between cardiac arrest and MRI (Sair et al., 2017), had limited (Koenig et al., 2014, Norton et al., 2012, Pugin et al., 2020) or skewed sample sizes (Sair et al., 2017), only included patients with indeterminate outcome (Koenig et al., 2014, Pugin et al., 2020), or contained high variability in timing of outcome definition, hampering extrapolation of results to early prediction. We found group differences within a group of prospectively enrolled patients, with MRI performed early and independent of clinical prediction of outcome at time of MRI. We were able to detect resting state networks in a group of patients with severe brain damage, highlighting the robustness of these networks. In addition, post hoc analyses show that the group differences remain significant after correction for currently used predictors of poor outcome, indicating a potential additional predictive value of functional MRI.

### 4.2 Regional vulnerability

We observed clear regional differences between networks in terms of disturbances of resting-state functional connectivity and mean diffusivity. There were networks where both functional connectivity and mean diffusivity consistently differed between the patients with good or poor outcome (large effect size for both measures, e.g. in the DMN), while other networks did not differ (low effect size for both measures, e.g. ECN). The largest effect sizes were found in the DMN and visual network, which are largely centred on the posterior side of the brain, whereas the smallest effects were in the ECN, which is located in the anterior part of the brain. This finding is consistent with previous fMRI studies, which have found most differences in the praecuneus and occipital areas between patients with good and poor outcome after cardiac arrest (Achard et al., 2012, Koenig et al., 2014, Wagner et al., 2020). This is in line with other studies showing relatively high ischemic vulnerability of posterior brain regions with other MRI modalities (Luigetti et al., 2012, Mlynash et al., 2010). Functional network hubs, identified by high levels of connectivity, tend to be metabolically more costly, e.g. due to higher glucose metabolism rates, compared to non-hubs (Bullmore and Sporns, 2012). These functional hubs are typically localized more posteriorly in the brain (Tomasi and Volkow, 2010). Thus, in case of metabolic distress like hypoxia after cardiac arrest, it is possible that these metabolically expensive networks are most sensitive to functional disruption. This is further substantiated by a study using PET scans in patients recovering from vegetative states, where a large increase of glucose metabolism was shown in the praecuneus (Laureys et al., 2006). Another factor contributing to the relative sensitivity to ischemia of posterior brain areas is the reduced density of sympathetic innervation in the posterior, compared to the anterior, circulation (Fugate and Rabinstein, 2015). Sympathetic innervation is essential for cerebral autoregulation (Buunk et al., 2000, ter Laan et al., 2013).

In the post hoc analysis including only patients who were comatose at the time of MRI, the difference between groups with poor and good outcome of connectivity strength in the visual network disappears.

### 4.3 The relationship between functional connectivity, diffusivity, and neurological outcome

MD within brain areas belonging to the large-scale networks identified with fMRI was reduced in patients with poor outcome, and this effect was largest in the DMN, SN, FPN, and visual network. This corresponds with networks that show highest group differences on fMRI. Where fMRI correlated with a good outcome, interindividual differences for MD were greatest for patients with poor outcome. This suggest that MD holds potential to contribute to prediction of poor outcome, which corresponds with previous studies on diffusion imaging after cardiac arrest (Hirsch et al., 2020, Keijzer et al., 2018, Keijzer et al., 2022, Vanden Berghe et al., 2020, Wouters et al., 2021), whereas fMRI seems better suited to predict a good outcome. Hence, functional connectivity measures and MD appear complimentary. This is further supported by post-hoc analyses showing that group differences in functional connectivity remained significant, within the DMN and the SN, in a subset of patients who all had an MD >650*10^−6^ mm^2^/s. The thresholds used here are based on eyeballing of the results and are only valid in this cohort. Therefore, these are preliminary in nature. However, the threshold of MD < 650*10^−6^ mm^2^/s is consistent with previous studies using ADC imaging (Hirsch et al., 2020, Keijzer et al., 2022, Wouters et al., 2021), where patients with diffusivity below this threshold also had a poor outcome. Our results indicate that it may be useful to combine functional connectivity and MD for prediction of outcome of patients in postanoxic coma. To determine clinically relevant thresholds, ROC analyses within separate training and validation sets are required. This will be tested in future research. Since only six out of the 48 included patients were in the indeterminate category of all three networks, a combination of multiple networks likely improves predictive values compared to the use of a single resting state network.

### 4.4 Pathophysiological considerations

BOLD fluctuations depend on fluctuations of cerebral blood flow, which in turn depend on fluctuations of brain activity (Attwell et al., 2010). Hereby, increases in cerebral perfusion are driven by vasoactive substances that are released on the postsynaptic side during synaptic transmission (Ekstrom, 2010, Tyler and Likova). Hence, increases of the BOLD response can be taken as an indirect measure of synaptic activity, especially with relatively normal MD. This interpretation fits with findings from experimental studies, which showed that disappearance of synaptic activity is a first consequence of global anoxia (Bolay et al., 2002, Hofmeijer and van Putten, 2012), and isolated synaptic failure may lead to permanent deficits in patients after cerebral ischemia (Hofmeijer and van Putten, 2012).

MD is an indirect but sensitive measure of structural neuronal and glial alterations like oedema. Prolonged ischaemia of the brain results in failure of active processes maintaining the resting membrane potential, such as the sodium potassium pumps. Failure of these processes results in an osmotic disbalance, causing an influx of water in the cell, and failure of action potential generation (Liang et al., 2007, Rungta et al., 2015).

These physiological correlates can explain the distribution of connectivity and MD values presented in this cohort. We find that patients with high connectivity and high MD show good neurological outcome, whereas low connectivity and low MD result in poor neurological outcome. This may indicate that intact synaptic functioning and absence of oedema predict a favourable outcome, and vice versa. Low functional connectivity with high MD may represent potential reversible synaptic failure in absence of oedema, indicating an indeterminate outcome. We found no patients showing a combination of high connectivity and low MD (Fig. 5). This would represent intact synaptic transmission, together with cytotoxic oedema, and is physiological impossible (Hofmeijer and van Putten, 2012).

### 4.5 The relationship between functional connectivity and EEG patterns

In general, favourable EEG categories were found in combination with high connectivity and unfavourable EEG categories in combination with low functional connectivity. However, there is a subgroup of patients with malignant EEG patterns but preserved connectivity, or vice versa. Our findings thus emphasize the potential additional value of EEG and fMRI, where EEG is sensitive to cortical activity, and fMRI to subcortical activity. The abundant EEG abnormalities in patients after cardiac arrest that were found in previous studies, point towards early synaptic failure (Ruijter et al., 2019), where typical EEG patterns likely reflect selective synaptic failure (Ruijter et al., 2017, Tjepkema-Cloostermans et al., 2014). Taken together, early recovery of synaptic functioning after cardiac arrest is reflected by relatively preserved functional connectivity in fMRI at day three, or early recovery of continuous EEG activity. This preserved integrity of largescale brain networks is associated with good long-term neurological recovery.

### 4.6 Clinical implications

Before we can make the step towards potential predictive values of functional connectivity for individual patients, a method should be established to estimate functional connectivity without the need for a group ICA. Templates of various networks, including the DMN and salience network, are available and could be of use for detection of resting state networks for individual patients (Smith et al., 2009, Yeo et al., 2011). However, these templates are based on healthy brains, without comorbidities. To facilitate future studies on resting state networks in our patient category, we added the templates of the resting state networks found here publicly available at Neurovault (https://neurovault.org/collections/12651/). Normal values and cut-off values predicting good or poor outcome, as calculated in this work, have to be validated in an independent cohort.

The additional value of the combination of structural and functional imaging and EEG to either of these measures alone should be validated. In this cohort, we had no patients that were indicated to have a good outcome by one test, but a poor outcome according to another test. For clinical use, validation of a multimodal algorithm should incorporate this possibility and indicate its clinical consequences.

Furthermore, performing fMRI and DTI scanning in comatose patients is a straining process that requires time and resources from the ICU and radiology department. To optimize the use of MRI measures, patients that will benefit from fMRI and DTI scanning should be identified. The EEG at 12 and 24 h will be able to identify those patients who are unlikely to recover (Bevers et al., 2018, Duez et al., 2019, Ruijter et al., 2019). For the remaining patients the prognosis is uncertain, fMRI and DTI scanning may potentially help in clinical decision making.

## 5 Strengths and limitations

Strengths of the study include the prospective design, the early timing of the MRI scans, and the use of hypothesis free data-driven analyses (ICA). To our knowledge, we are the first to combine functional connectivity and MD within a multimodal approach. There are also some limitations: although our sample (n = 48) is decent when compared to previous fMRI studies in the field (which included only 12–17 patients (Achard et al., 2012, Koenig et al., 2014, Norton et al., 2012)), the sample size is still relatively small when moving towards cut-off values to be used in individual prognosis. Although we found highly significant group effects, a larger cohort with an independent validation sample will be needed to establish predictive values for the individual patient. Second, study site remained a significant confounder in the fMRI analyses, even after carefully harmonizing the MRI protocols. Third, the influence of a self-fulfilling prophecy cannot be fully excluded, which plagues literature on outcome prediction after cardiac arrest in general. Although, decisions on WLST were never based on brain MRI, factors that likely correlate with MRI, such as EEG background, co-morbidities, clinical severity, etc. were included in this decision. Fourth, given that our goal was to detect differences between patients with a poor versus good outcome, we did not include a healthy control group. Hence, we are unable to directly compare the networks between patients and controls. Finally, sedation may have affected the results: 34 % of patients with a good outcome were sedated, whereas this was 89 % of patients with a poor outcome. We decided not to correct for the effect of sedation, because the level of sedation is associated with the outcome of the patients. Comatose patients with ultimately favourable recovery typically need higher levels of sedation than patients with poor outcome. On the other hand, a subgroup of patients with predominantly good outcome was already extubated at day 3, and required no sedation. Resting-state networks have been persistently observed under light (humans) (Greicius et al., 2008) and deep (primates) (Vincent et al., 2007) anaesthesia, indicating that the effect of sedation on intrinsic functional connectivity within resting state networks may not be large. In future larger cohorts, subgroup analyses in patients with comparable levels of sedation, who have a good versus poor neurological outcome, would be of value.

## 6 Conclusion

Preserved resting-state functional connectivity and high mean diffusivity in almost all resting state networks at day three after cardiac arrest is associated with good neurological recovery-six months later. These effects are largest for networks centred on posterior brain regions, e.g. the DMN and visual network. The combination of fMRI and DTI holds potential to improve discrimination of comatose patients with good and poor outcome after cardiac arrest.

## Declaration of Competing Interest

The authors declare that they have no known competing financial interests or personal relationships that could have appeared to influence the work reported in this paper.

## Data Availability

Anonymized data can be made available to other researchers upon request, one year after completion of the Cracking Coma study.
