# Supplementary material for: MRI markers of brain network integrity relate to neurological outcome in postanoxic coma
Source: Neuroimage Clin. 2022 Aug 26;36:103171. doi: 10.1016/j.nicl.2022.103171 (PMC9446009; doi:10.1016/j.nicl.2022.103171)
Supplement: Supplementary data 1 [file mmc1.docx]

Supplementary Material:

MRI markers of brain network integrity relate to neurological outcome in postanoxic coma.

Keijzer et al

Table of contents

[Detailed overview of fMRIprep preprocessing 2](#_Toc111717098)

[Supplementary figure 1. Overview of screening and enrolment 4](#_Toc111717099)

[Supplementary table 1: group statistics (ANCOVA) of functional connectivity per network 5](#_Toc111717100)

[Supplementary table 2: group statistics (ANCOVA) of mean diffusivity per network 5](#_Toc111717101)

[Supplementary table 3: group statistics (ANCOVA) of functional connectivity for comatose patients at time of MRI 6](#_Toc111717102)

[Supplementary table 4: group effects of current predictors of poor outcome in addition to functional connectivity (ANCOVA) 6](#_Toc111717103)

[Supplementary table 5: group comparisons of mean normalized connectivity in subset of patients with MD > threshold 6](#_Toc111717104)

[References 7](#_Toc111717105)

# Detailed overview of fMRIprep preprocessing

Results included in this manuscript come from preprocessing performed using fMRIPrep 20.0.6 (Esteban, Markiewicz, et al. (2018); Esteban, Blair, et al. (2018); RRID:SCR_016216), which is based on Nipype 1.4.2 (Gorgolewski et al. (2011); Gorgolewski et al. (2018); RRID:SCR_002502).

1.1 Anatomical data preprocessing

    A total of 3 T1-weighted (T1w) images were found within the input BIDS dataset. All of them were corrected for intensity non-uniformity (INU) with N4BiasFieldCorrection (Tustison et al. 2010), distributed with ANTs 2.2.0 (Avants et al. 2008, RRID:SCR_004757). The T1w-reference was then skull-stripped with a Nipype implementation of the antsBrainExtraction.sh workflow (from ANTs), using OASIS30ANTs as target template. Brain tissue segmentation of cerebrospinal fluid (CSF), white-matter (WM) and gray-matter (GM) was performed on the brain-extracted T1w using fast (FSL 5.0.9, RRID:SCR_002823, Zhang, Brady, and Smith 2001). A T1w-reference map was computed after registration of 3 T1w images (after INU-correction) using mri_robust_template (FreeSurfer 6.0.1, Reuter, Rosas, and Fischl 2010). Brain surfaces were reconstructed using recon-all (FreeSurfer 6.0.1, RRID:SCR_001847, Dale, Fischl, and Sereno 1999), and the brain mask estimated previously was refined with a custom variation of the method to reconcile ANTs-derived and FreeSurfer-derived segmentations of the cortical gray-matter of Mindboggle (RRID:SCR_002438, Klein et al. 2017). Volume-based spatial normalization to two standard spaces (MNI152NLin2009cAsym, MNI152NLin6Asym) was performed through nonlinear registration with antsRegistration (ANTs 2.2.0), using brain-extracted versions of both T1w reference and the T1w template. The following templates were selected for spatial normalization: ICBM 152 Nonlinear Asymmetrical template version 2009c [Fonov et al. (2009), RRID:SCR_008796; TemplateFlow ID: MNI152NLin2009cAsym], FSL\u2019s MNI ICBM 152 non-linear 6th Generation Asymmetric Average Brain Stereotaxic Registration Model [Evans et al. (2012), RRID:SCR_002823; TemplateFlow ID: MNI152NLin6Asym],

1.2 Functional data preprocessing

    For each of the 3 BOLD runs found per subject (across all tasks and sessions), the following preprocessing was performed. First, a reference volume and its skull-stripped version were generated using a custom methodology of fMRIPrep. Susceptibility distortion correction (SDC) was omitted. The BOLD reference was then co-registered to the T1w reference using bbregister (FreeSurfer) which implements boundary-based registration (Greve and Fischl 2009). Co-registration was configured with six degrees of freedom. Head-motion parameters with respect to the BOLD reference (transformation matrices, and six corresponding rotation and translation parameters) are estimated before any spatiotemporal filtering using mcflirt (FSL 5.0.9, Jenkinson et al. 2002). The BOLD time-series (including slice-timing correction when applied) were resampled onto their original, native space by applying the transforms to correct for head-motion. These resampled BOLD time-series will be referred to as preprocessed BOLD in original space, or just preprocessed BOLD. The BOLD time-series were resampled into standard space, generating a preprocessed BOLD run in MNI152NLin2009cAsym space. First, a reference volume and its skull-stripped version were generated using a custom methodology of fMRIPrep. Automatic removal of motion artifacts using independent component analysis (ICA-AROMA, Pruim et al. 2015) was performed on the preprocessed BOLD on MNI space time-series after removal of non-steady state volumes and spatial smoothing with an isotropic, Gaussian kernel of 6mm FWHM (full-width half-maximum). Corresponding \u201cnon-aggresively\u201d denoised runs were produced after such smoothing. Additionally, the \u201caggressive\u201d noise-regressors were collected and placed in the corresponding confounds file. Several confounding time-series were calculated based on the preprocessed BOLD: framewise displacement (FD), DVARS and three region-wise global signals. FD and DVARS are calculated for each functional run, both using their implementations in Nipype (following the definitions by Power et al. 2014). The three global signals are extracted within the CSF, the WM, and the whole-brain masks. Additionally, a set of physiological regressors were extracted to allow for component-based noise correction (CompCor, Behzadi et al. 2007). Principal components are estimated after high-pass filtering the preprocessed BOLD time-series (using a discrete cosine filter with 128s cut-off) for the two CompCor variants: temporal (tCompCor) and anatomical (aCompCor). tCompCor components are then calculated from the top 5% variable voxels within a mask covering the subcortical regions. This subcortical mask is obtained by heavily eroding the brain mask, which ensures it does not include cortical GM regions. For aCompCor, components are calculated within the intersection of the aforementioned mask and the union of CSF and WM masks calculated in T1w space, after their projection to the native space of each functional run (using the inverse BOLD-to-T1w transformation). Components are also calculated separately within the WM and CSF masks. For each CompCor decomposition, the k components with the largest singular values are retained, such that the retained components\u2019 time series are sufficient to explain 50 percent of variance across the nuisance mask (CSF, WM, combined, or temporal). The remaining components are dropped from consideration. The head-motion estimates calculated in the correction step were also placed within the corresponding confounds file. The confound time series derived from head motion estimates and global signals were expanded with the inclusion of temporal derivatives and quadratic terms for each (Satterthwaite et al. 2013). Frames that exceeded a threshold of 0.5 mm FD or 1.5 standardised DVARS were annotated as motion outliers. All resamplings can be performed with a single interpolation step by composing all the pertinent transformations (i.e. head-motion transform matrices, susceptibility distortion correction when available, and co-registrations to anatomical and output spaces). Gridded (volumetric) resamplings were performed using antsApplyTransforms (ANTs), configured with Lanczos interpolation to minimize the smoothing effects of other kernels (Lanczos 1964). Non-gridded (surface) resamplings were performed using mri_vol2surf (FreeSurfer).

Many internal operations of fMRIPrep use Nilearn 0.6.2 (Abraham et al. 2014, RRID:SCR_001362), mostly within the functional processing workflow. For more details of the pipeline, see the section corresponding to workflows in fMRIPrep\u2019s documentation.

1.3 Copyright Waiver

The above boilerplate text was automatically generated by fMRIPrep with the express intention that users should copy and paste this text into their manuscripts unchanged. It is released under the CC0 license.

# Supplementary figure 1. Overview of screening and enrolment


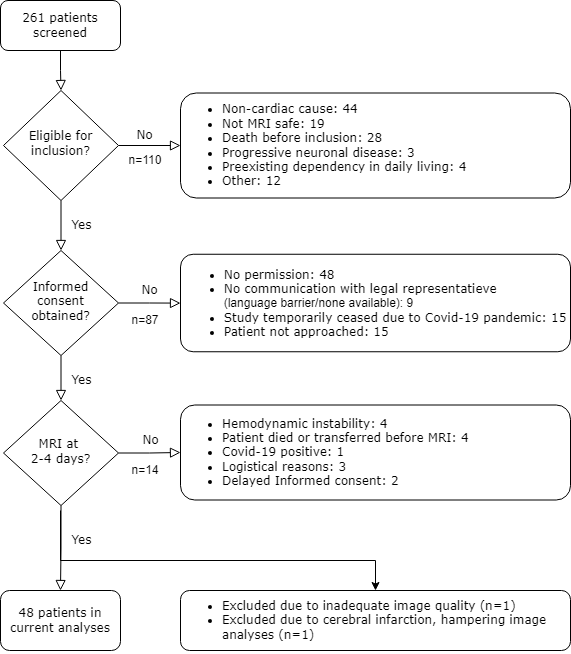


##

# Supplementary table 1: group statistics (ANCOVA) of functional connectivity per network

| Network | Mean z-score (± sd) | | p-value* | Cohens d | Effect study site | Effect FD |
| --- | --- | --- | --- | --- | --- | --- |
|  | Good (n=29) | Poor (n=19) |  |  |  |  |
| DMN | 3.51 ± 1.29 | 2.09 ± 0.90 | <0.01 | 1.23 | 0,06 | 0,72 |
| Salience | 2.88 ± 0.66 | 2.05 ± 0.58 | <0.01 | 1.31 | 0,01 | 0,07 |
| FPN | 2.78 ± 0.90 | 1.96 ± 0.92 | 0.02 | 0.91 | 0,04 | 0,68 |
| ECN | 2.63 ± 1.78 | 2.07 ± 0.50 | 0.56 | 0.58 | 0,16 | 0,52 |
| Visual | 4.49 ± 1.29 | 2.83 ± 1.14 | <0.01 | 1.34 | 0,04 | 0,68 |
| Sensorimotor | 2.36 ± 0.72 | 1.86 ± 0.45 | 0.02 | 0.80 | 0,03 | 0,14 |
| Reward | 3.23 ± 1.63 | 2.27 ± 0.52 | 0.02 | 0.73 | 0,01 | 0,88 |
| Auditory | 3.35 ± 1.33 | 2.37 ± 0.74 | 0.02 | 0.87 | 0,04 | 0,61 |
| Motor | 2.65 ± 0.79 | 2.13 ± 0.90 | 0.06 | 0.63 | 0,02 | 0,62 |
| Cerebellar | 3.95 ± 1.78 | 2.73 ± 0.85 | <0.01 | 0.82 | 0,01 | 0,44 |

*DMN: Default mode network, ECN: Executive control network, FD: Framewise displacement, FPN: frontoparietal network
*P-values are corrected for study site and framewise displacement. Correction for multiple comparisons was applied using the false discovery rate*

# Supplementary table 2: group statistics (ANCOVA) of mean diffusivity per network

| Network | Mean (± sd)* | | p-value** | Cohens d | Effect study site |
| --- | --- | --- | --- | --- | --- |
|  | Good (n=29) | Poor (n=19) |  |  |  |
| DMN | 0,71 ± 0.03 | 0,61 | <0.01 | 1,32 | 0,04 |
| Salience | 0,72 ± 0.03 | 0,63 | <0.01 | 1,06 | 0,05 |
| FPN | 0,71 ± 0.03 | 0,62 | <0.01 | 1,23 | 0,03 |
| ECN | 0,68 ± 0.02 | 0,63 | 0.01 | 0,77 | 0,09 |
| Visual | 0,69 ± 0.04 | 0,55 | <0.01 | 1,69 | 0,01 |
| Sensorimotor | 0,73 ± 0.04 | 0,62 | <0.01 | 1,48 | 0,09 |
| Reward | 0,69 ± 0.03 | 0,63 | <0.01 | 0,83 | 0,09 |
| Auditory | 0,69 ± 0.03 | 0,60 | <0.01 | 1,16 | 0,04 |
| Motor | 0,70 ± 0.04 | 0,60 | <0.01 | 1,37 | 0,10 |
| Cerebellar | 0,60 ± 0.03 | 0,56 | <0.01 | 0,78 | 0,03 |

*DMN: Default mode network, ECN: Executive control network, FPN: frontoparietal network
* MD x10^-3^ mm^2^/s
**P-values are corrected for study site and framewise displacement. Correction for multiple comparisons was applied using the false discovery rate*

# Supplementary table 3: group statistics (ANCOVA) of functional connectivity for comatose patients at time of MRI

| Network | Mean (± sd) | | p-value* | Cohens d |
| --- | --- | --- | --- | --- |
|  | Good (n=10) | Poor (n=18) |  |  |
| DMN | 2.80 ± 0.87 | 1.95 ± 0.69 | 0.01 | 1.13 |
| Salience | 2.61 ± 0.72 | 2.01 ± 0.56 | <0.01 | 0.98 |
| visual | 3.58 ± 1.05 | 2.74 ± 1.10 | 0.06 | 0.30 |

*DMN: Default mode network
*P-values are corrected for study site and framewise displacement.*

# Supplementary table 4: group effects of current predictors of poor outcome in addition to functional connectivity (ANCOVA)

| Network | Effect* | | | | | | |
| --- | --- | --- | --- | --- | --- | --- | --- |
|  | Connectivity | FD | Study site | EEG | SSEP | PR | CR |
| DMN | 0.03 | 0.57 | 0.13 | 0.11 | 0.24 | 0.38 | 0.27 |
| Salience | <0.01 | 0.10 | 0.04 | 0.14 | 0.68 | 0.28 | 0.27 |
| visual | 0.01 | 0.96 | 0.11 | 0.14 | 0.46 | 0.19 | 0.38 |

*DMN: Default mode network; FD: framewise displacement, SSEP: bilaterally absent somatosensory evoked potentials (N20); PR: pupillary reflex, CR: corneal reflex.
* An effect of p<0.05 is considered significant*

# Supplementary table 5: group comparisons of mean normalized connectivity in subset of patients with MD > threshold

| **Network** | **Minimal MD** | **Subset size / good outcome (n/n)** | **Good outcome**  **Median [IQR]** | **Poor outcome**  **Median [IQR]** | **p-value** |
| --- | --- | --- | --- | --- | --- |
| DMN | 650 | 38/29 | 3.4 [2.7-4.3] | 1.9 [1.9-3.6] | 0.04 |
| Salience | 650 | 40/29 | 2.9 [2.5-3.7] | 1.9 [1.8-2.4] | <0.01 |
| Visual | 600 | 36/29 | 4.7 [3.6-5.1] | 3.7 [2.5-4.3] | 0.32 |

# References

Abraham, Alexandre, Fabian Pedregosa, et al. 2014. Machine Learning for Neuroimaging with Scikit-Learn. Frontiers in Neuroinformatics 8. <https://doi.org/10.3389/fninf.2014.00014>.

Avants, B.B., C.L. Epstein, M. Grossman, and J.C. Gee. 2008. Symmetric Diffeomorphic Image Registration with Cross-Correlation: Evaluating Automated Labeling of Elderly and Neurodegenerative Brain. Medical Image Analysis 12 (1): 26. <https://doi.org/10.1016/j.media.2007.06.004>.

Behzadi, Yashar, Khaled Restom, Joy Liau, and Thomas T. Liu. 2007. A Component Based Noise Correction Method (CompCor) for BOLD and Perfusion Based fMRI. NeuroImage 37 (1): 90. <https://doi.org/10.1016/j.neuroimage.2007.04.042>.

Dale, Anders M., Bruce Fischl, and Martin I. Sereno. 1999. Cortical Surface-Based Analysis: I. Segmentation and Surface Reconstruction. NeuroImage 9 (2): 179. <https://doi.org/10.1006/nimg.1998.0395>.

Esteban, Oscar, Ross Blair, Christopher et al. 2018. FMRIPrep.Software. Zenodo. <https://doi.org/10.5281/zenodo.852659>.

Esteban, Oscar, Christopher Markiewicz, Ross W Blair, et al. 2018. fMRIPrep: A Robust Preprocessing Pipeline for Functional MRI. Nature Methods. <https://doi.org/10.1038/s41592-018-0235-4>.

Evans, AC, AL Janke, DL Collins, and S Baillet. 2012. Brain Templates and Atlases. NeuroImage 62 (2): 911. <https://doi.org/10.1016/j.neuroimage.2012.01.024>.

Fonov, VS, AC Evans, RC McKinstry, CR Almli, and DL Collins. 2009. Unbiased Nonlinear Average Age-Appropriate Brain Templates from Birth to Adulthood. NeuroImage 47, Supplement 1: S102. <https://doi.org/10.1016/S1053-8119(09)70884-5>.

Gorgolewski, K., C. D. Burns, C. Madison, D. Clark, Y. O. Halchenko, M. L. Waskom, and S. Ghosh. 2011. Nipype: A Flexible, Lightweight and Extensible Neuroimaging Data Processing Framework in Python. Frontiers in Neuroinformatics 5: 13. <https://doi.org/10.3389/fninf.2011.00013>.

Gorgolewski, Krzysztof J., Oscar Esteban, Christopher J. et al. 2018. Nipype. Software. Zenodo. <https://doi.org/10.5281/zenodo.596855>.

Greve, Douglas N, and Bruce Fischl. 2009. Accurate and Robust Brain Image Alignment Using Boundary-Based Registration. NeuroImage 48 (1): 63. <https://doi.org/10.1016/j.neuroimage.2009.06.060>.

Jenkinson, Mark, Peter Bannister, Michael Brady, and Stephen Smith. 2002. Improved Optimization for the Robust and Accurate Linear Registration and Motion Correction of Brain Images. NeuroImage 17 (2): 825. <https://doi.org/10.1006/nimg.2002.1132>.

Klein, Arno, Satrajit S. Ghosh, Forrest S. Bao, Joachim Giard, Yrjö Häme, Eliezer Stavsky, Noah Lee, et al. 2017. Mindboggling Morphometry of Human Brains. PLOS Computational Biology 13 (2): e1005350. <https://doi.org/10.1371/journal.pcbi.1005350>.

Lanczos, C. 1964. Evaluation of Noisy Data. Journal of the Society for Industrial and Applied Mathematics Series B Numerical Analysis 1 (1): 76. <https://doi.org/10.1137/0701007>.

Power, Jonathan D., Anish Mitra, Timothy O. Laumann, et al. 2014. Methods to Detect, Characterize, and Remove Motion Artifact in Resting State fMRI. NeuroImage 84 (Supplement C): 320. <https://doi.org/10.1016/j.neuroimage.2013.08.048>.

Pruim, Raimon H. R., Maarten Mennes, Daan van Rooij, Alberto Llera, Jan K. Buitelaar, and Christian F. Beckmann. 2015. ICA-AROMA: A Robust ICA-Based Strategy for Removing Motion Artifacts from fMRI Data. NeuroImage 112 (Supplement C): 267. <https://doi.org/10.1016/j.neuroimage.2015.02.064>.

Reuter, Martin, Herminia Diana Rosas, and Bruce Fischl. 2010. Highly Accurate Inverse Consistent Registration: A Robust Approach. NeuroImage 53 (4): 1181. <https://doi.org/10.1016/j.neuroimage.2010.07.020>.

Satterthwaite, Theodore D., Mark A. Elliott, Raphael T., et al. 2013. An improved framework for confound regression and filtering for control of motion artifact in the preprocessing of resting-state functional connectivity data. NeuroImage 64 (1): 240. <https://doi.org/10.1016/j.neuroimage.2012.08.052>.

Tustison, N. J., B. B. Avants, P. A. Cook, Y. Zheng, A. Egan, P. A. Yushkevich, and J. C. Gee. 2010. N4ITK: Improved N3 Bias Correction. IEEE Transactions on Medical Imaging 29 (6): 1310. <https://doi.org/10.1109/TMI.2010.2046908>.

Zhang, Y., M. Brady, and S. Smith. 2001. Segmentation of Brain MR Images Through a Hidden Markov Random Field Model and the Expectation-Maximization Algorithm. IEEE Transactions on Medical Imaging 20 (1): 45. <https://doi.org/10.1109/42.906424>.
